# Daratumumab in the Treatment of Light-Chain (AL) Amyloidosis

**DOI:** 10.3390/cells10030545

**Published:** 2021-03-04

**Authors:** Giovanni Palladini, Paolo Milani, Fabio Malavasi, Giampaolo Merlini

**Affiliations:** 1 Amyloidosis Research and Treatment Center, Foundation “Istituto di Ricovero e Cura a Carattere Scientifico (IRCCS) Policlinico San Matteo”, 27100 Pavia, Italy; giovanni.palladini@unipv.it (G.P.); paolomilani.pv@gmail.com (P.M.); 2 Department of Molecular Medicine, University of Pavia, 10121 Pavia, Italy; 3 Laboratory of Immunogenetics, Department of Medical Sciences, Center for Experimental Research and Medical Studies (CeRMS), University of Turin, Fondazione Ricerca Molinette, 10121 Turin, Italy; fabio.malavasi@unito.it

**Keywords:** light-chain amyloidosis, CD38, monoclonal antibody, immunotherapy

## Abstract

Systemic light-chain (AL) amyloidosis is caused by a small B cell, most commonly a plasma cell (PC), clone that produces toxic light chains (LC) that cause organ dysfunction and deposits in tissues. Due to the production of amyloidogenic, misfolded LC, AL PCs display peculiar biologic features. The small, indolent plasma cell clone is an ideal target for anti-CD38 immunotherapy. A recent phase III randomized study showed that in newly diagnosed patients, the addition of daratumumab to the standard of care increased the rate and depth of the hematologic response and granted more frequent organ responses. In the relapsed/refractory setting, daratumumab alone or as part of combination regimens gave very promising results. It is likely that daratumumab-based regimens will become new standards of care in AL amyloidosis. Another anti-CD38 monoclonal antibody, isatuximab, is at an earlier stage of development as a treatment for AL amyloidosis. The ability to target CD38 on the amyloid PC offers new powerful tools to treat AL amyloidosis. Future studies should define the preferable agents to combine with daratumumab upfront and in the rescue setting and assess the role of maintenance. In this review, we summarize the rationale for using anti-CD38 antibodies in the treatment of AL amyloidosis.

## 1. Introduction

Light-chain (AL) amyloidosis is caused by a small B cell clone producing an immunoglobulin light chain that causes target organ toxicity and deposits in tissues in the form of amyloid fibrils [1]. The clinical presentation and outcome of AL amyloidosis are determined by organ involvement [2]. The most commonly involved organs are the heart and kidneys, and patients often present with heart failure and nephrotic syndrome. Patients with advanced cardiac amyloidosis are at the highest risk of early death, with a median survival of a few months. The liver and peripheral and autonomic nervous systems can also be involved, resulting in cholestasis, peripheral neuropathy, hypotension, and diarrhea or constipations. This makes AL amyloidosis a hematologic malignancy associated with significant multiorgan dysfunction and patient frailty, resulting in specific difficulties, and requiring caution in treatment delivery. Thus, the treatment strategy needs to be risk-adapted based on the severity of organ dysfunction, age, and comorbidities [3].

The current treatment of AL amyloidosis targets the underlying clonal disease to reduce the supply of the amyloid light chain [3]. Other approaches targeting the amyloid deposits are under investigation [4]. It is largely recognized that rapid and deep reductions in the circulating amyloid light chains are key to improve organ dysfunction and extend survival [5]. This is true also in high-risk patients [6]. Thus, treatment of AL amyloidosis needs to be response-adapted, and patients should be followed closely during chemotherapy, in order to detect early those who fail to reach a satisfactory hematologic response and promptly start a rescue therapy. Response criteria based on serum and urine immunofixation and measurement of circulating free light chains have been validated based on survival outcomes [7,8,9].

## 2. The Amyloid Clone 

In the vast majority of cases, the amyloid clone is composed of plasma cells (PCs), and agents and regimens used in multiple myeloma are also employed in AL amyloidosis. In a smaller subset of patients, the amyloid clone has the characteristics of a marginal zone lymphoma or a lymphoplasmacytic clone [10,11,12]. The amyloid plasma cell clone is smaller than that of multiple myeloma [1,13], with a median bone marrow infiltrate of only 10%. Amyloidogenic PCs display phenotypic and copy number alteration profiles similar to multiple myeloma (MM), but their transcriptome is remarkably similar to that of normal PCs [14,15]. The amyloid clone resembles monoclonal gammopathy of unknown significance (MGUS) in terms of copy number changes and driver gene mutations and lies within the continuous spectrum from MGUS to MM [16]. In addition, whole genome sequencing data revealed a lower mutation burden compared to myeloma clones, reflecting a stable genetic status of the disease from a mutational perspective [17]. Previous genome-wide association studies (GWASs) have identified specific loci for MM and six of these were related to AL amyloidosis. In addition, four single-nucleotide polymorphisms (SNPs), not related to MM, were significantly found [18]. It was reported that the cyclin D1 locus appeared as a more prominent driver for AL amyloidosis patients compared to MM and MGUS patients and it could offer possible new therapeutic targets [19].

This small clone is striving to counteract the intrinsic cellular stress produced by misfolded light chains. It has been shown that amyloidogenic light chain (LC) production is associated with distinctive organellar feature and expression patterns indicative of cellular stress [20]. These consisted of an expanded endoplasmic reticulum, perinuclear mitochondria, and a higher abundance of stress-related transcripts and were consistent with reduced autophagic control of organelle homeostasis. Therefore, the amyloid plasma cells rely on proteasome activity to control the toxicity of the light chain they produce [20]. This makes the amyloid clone particularly sensitive to proteasome inhibition, and bortezomib is now largely used in the upfront therapy of patients with AL amyloidosis [3]. However, recent studies showed that the most common (>50% of patients) chromosomal abnormality found in the amyloid plasma cell clone, t(11;14), is associated with less frequent and less deep hematologic responses and shorter survival when bortezomib is used in combination with dexamethasone and/or cyclophosphamide [21,22,23,24,25]. Novel agents with different mechanisms of action are necessary to improve the therapy outcome. The reduced size of the amyloid clone makes it an ideal target of immunotherapies directed to the plasma cells. As expected, amyloidogenic plasma cells express on the surface CD38, a type 2 transmembrane ectoenzyme, whose molecular, structural, and functional features are exhaustively described in [26]. CD38 is a multifunctional protein that acts as both a receptor and an ectoenzyme, involved in cellular and tissue NAD+ homeostasis, and also in the generation of the second messengers adenosine diphosphoribose (ADPR) and cyclic-ADPR (cADPR) that are essential for intracellular calcium signaling. Furthermore, the initial disassembly of NAD+ is also followed by adenosinergic activity, if CD38 is operating in the presence of CD203a and CD73 nucleotidases. Signaling adenosine is tolerogenic in the MM niche, contributing to the immunosurveillance escape of myeloma cells [27]. This is clinically relevant since the anti-CD38 antibody isatuximab inhibits the enzymatic activity of CD38 [28].

Higher CD38 expression in AL amyloidosis is associated with the worst event-free survival probably because it is associated with more severe cardiac involvement, as shown by higher serum NT-proBNP concentrations [29].

## 3. Daratumumab

The first anti-CD38 monoclonal antibody becoming available in multiple myeloma was daratumumab, and in a couple of years, a few prospective trials and many retrospective studies assessed its role also in AL amyloidosis. Daratumumab is a fully human IgG1κ monoclonal antibody directed against CD38 that acts both as a PC-depleting agent and as an immuno-modulator [30]. Engagement of CD38 leads to apoptosis of plasma cells through Fc-mediated cross-linking, and immune-mediated tumor cell lysis through complement- and antibody-dependent cytotoxicity and phagocytosis. Daratumumab works not only on the tumor target, but also on the immune effectors, eliminating immunosuppressive populations such as T regulatory lymphocytes, B regulatory lymphocytes, and myeloid-derived suppressor cells, while T cell populations are increased [31]. Daratumumab targeting of CD38+ NK cells may play a pivotal role in initiating a Th1-mediated immune response, which can be an essential component in mounting a powerful anti-CD38 immune response against myeloma cells [32]. Furthermore, daratumumab induces a redistribution of CD38, together with morphological modifications, leading to release of antibody-covered microvesicles which are selectively captured and internalized by cells expressing Fc receptors (FcRs), such as monocytes, myeloid-derived suppressor cells, and NK cells, modulating their activity. The possibility that such microvesicles may interact with dendritic cells producing vaccination-related effects, as reported with isatuximab [33], is under investigation.

## 4. In Vivo Applications

Daratumumab is labeled for the treatment of newly diagnosed and relapsed refractory MM patients and is available for both intravenous and subcutaneous injections. On 15 January 2021, the US Food and Drug Administration (FDA) granted accelerated approval to daratumumab and hyaluronidase-fihj in combination with bortezomib, cyclophosphamide, and dexamethasone (DARA-CyBorD) for the treatment of newly diagnosed adult patients with light-chain (AL) amyloidosis [34].

### 4.1. Daratumumab in the First-Line Treatment of Patients with AL Amyloidosis

**Figure 1:**
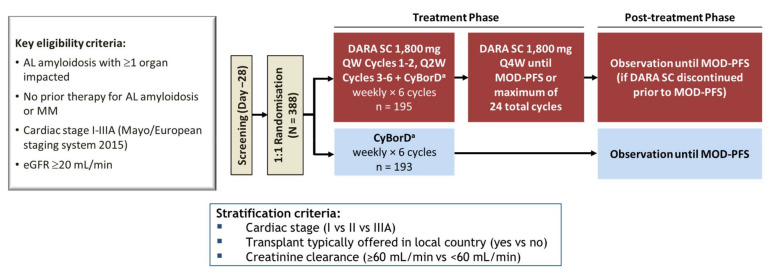
ANDROMEDA study design. Legend: CyBorD, cyclophosphamide, bortezomib, and dexamethasone; DARA, daratumumab; eGFR, estimated glomerular filtration rate; IV, intravenous; QW, weekly; Q2W, twice weekly; Q4W, every 4 weeks; MOD-PFS, major organ deterioration progression-free survival; PO, per os; SC, subcutaneous. Patients received dexamethasone 20 mg on the day of DARA SC dosing and 20 mg on the day after DARA dosing.

The role of daratumumab in upfront treatment of patients with AL amyloidosis was explored in the recently completed randomized phase III ANDROMEDA trial (NCT03201965). The randomized part of this trial was preceded by a safety run-in study to assess the tolerability of the addition of subcutaneous daratumumab to CyBorD [35]. The treatment schedule of daratumumab-CyBorD is reported in Figure 1. The safety run-in study included 28 patients, 27 of whom attained a hematologic response (96%), which was very good partial response (VGPR) or complete response (CR) in 82% of the cases [35]. Remarkably, the time to first hematologic response (median 9 days) and to deep response (median 19 days) was exceptionally short. Such a rapid response is very relevant for patients with AL amyloidosis who need a rapid reduction in the toxic light chain to improve target organ dysfunction and extend survival [2]. The rate of organ response was also very high, with improvement of cardiac and renal involvement in 53% and 83% of patients, respectively [35]. These very promising results were confirmed in the larger randomized portion of the ANDROMEDA study [36]. A total of 388 patients were randomized to receive daratumumab-CyBorD (195 subjects) or CyBorD alone (193 subjects). Importantly, in the CyBorD arm, treatment was discontinued after six cycles, whereas, in the experimental arm, after six courses of daratumumab-CyBorD, daratumumab alone was administered monthly for 2 years or until progression. Second-line therapy was initiated in 42% of patients in the CyBorD arm and in 10% of subjects in the daratumumab-CyBorD arm. Patients with advanced cardiac involvement were excluded from this trial. The overall hematologic response rate (92% vs. 77%), as well as the rate of VGPR/CR (79% vs. 49%), was higher in the daratumumab arm. Moreover, rates of cardiac and renal responses at 6 months were significantly higher with daratumumab-CyBorD (42% vs. 22% and 54% vs. 27%, respectively). In addition, in the ANDROMEDA trial, a novel time-to-event endpoint, major organ deterioration progression-free survival (MOD PFS), defined as the time to end-stage cardiac or renal disease or death or hematologic progression, was used to assess treatment efficacy. When considering PFS, one should keep in mind that currently in AL amyloidosis, we lack validated criteria for hematologic progression which are based on a consensus statement [37]. In the ANDROMEDA study, earlier and deeper hematologic responses were associated with prolonged MOD PFS [38,39], and MOD PFS was longer with daratumumab-CyBorD than CyBorD alone. With a median follow-up of 11 months, MOD PFS is mainly driven by hematologic progression and a longer observation will be needed to assess the impact of the addition of daratumumab and of maintenance therapy on time-to-event endpoints.

Hematologic and organ responses are very strong predictors of overall survival in AL amyloidosis, and these unprecedentedly high response rates strongly support daratumumab-CyBorD as a novel standard of care for upfront treatment of patients with this disease [3]. Moreover, the very short time to deep hematologic response makes daratumumab an appealing option for patients with advanced disease, and a phase II study of single-agent subcutaneous daratumumab in this setting is underway (NCT04131309).

### 4.2. Daratumumab in the Treatment of Relapsed/Refractory Patients with AL Amyloidosis

**Figure 2:**
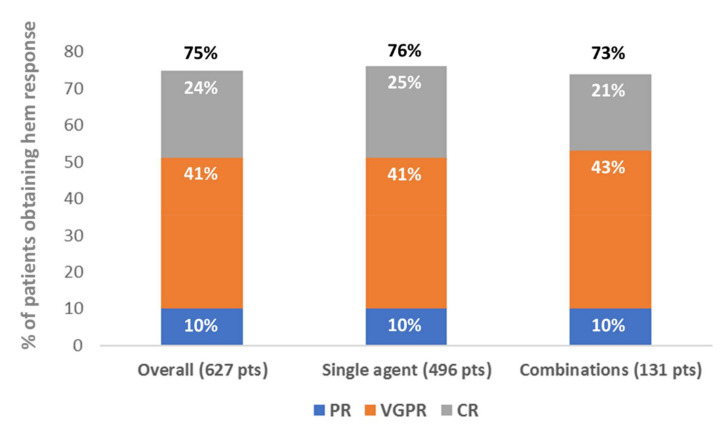
Overall response rate of the retrospective studies on daratumumab in light-chain (AL) amyloidosis. Legend: CR, complete response; PR, partial response; VGPR, very good partial response.

Two phase II clinical trials evaluated daratumumab as a rescue therapy for patients with AL amyloidosis [40,41]. Moreover, once daratumumab became available for multiple myeloma, many relapsed/refractory patients with AL amyloidosis had access to this drug as well, and almost 20 case reports and retrospective series were published in 4 years [42,43,44,45,46,47,48,49,50,51,52,53,54,55,56,57,58,59,60]. Differently from the frontline trial that evaluated subcutaneous daratumumab, the intravenous drug was employed in these studies. Figure 2 summarizes the response rates with daratumumab combinations in published patients with relapsed/refractory AL amyloidosis and Table 1 reports a comparison of the published retrospective series.

In a phase II prospective trial, Sanchorawala et al. treated 22 patients with previously treated AL amyloidosis with single-agent daratumumab [40]. Overall, 96% of patients attained a hematologic response, which was VGPR or CR in 86% of patients. Remarkably, in three patients, minimal residual disease was not detectable by multiparameter flow cytometry. Cardiac and renal responses were observed in 50% and 67% of patients. In a European phase II trial, Roussel et al. offered single-agent daratumumab to 40 relapsed/refractory patients [41]. Fifty-five percent of patients reached a hematologic response, which was VGPR in at least 48% of cases. Cardiac and renal responses were obtained in 25% and 31% of patients, respectively.

Retrospective studies of daratumumab in relapsed/refractory patients with AL amyloidosis also addressed daratumumab-based combinations. The two largest studies were published by the Heidelberg [56] and Pavia [57] referral centers. Kimmich et al. reported 168 consecutive patients treated with daratumumab and dexamethasone (106) or daratumumab, bortezomib, and dexamethasone (62) [56]. The overall hematologic response rate (64% and 66%) and VGPR/CR rate (48% and 55%) were similar with and without the addition of bortezomib, as well as the cardiac response (22% and 26%). Remarkably, t(11;14) was associated with a longer PFS in this series, whereas high differential FLC levels (>180 mg/L) and nephrotic range proteinuria were associated with poorer PFS. The authors speculated that the latter observation might be due to urinary loss of the monoclonal antibody, and this hypothesis deserves further investigation. Milani and coworkers reported 72 patients with relapsed/refractory AL amyloidosis and a baseline bone marrow plasma cell infiltrate of >10% treated with daratumumab single agent (47) or combined with bortezomib (14) or lenalidomide (11) [57]. The overall hematologic response rate was 83% (VGPR/CR 59%) and cardiac and renal responses were attained in 29% and 60% of patients, respectively. No difference was observed in overall (81% and 88%) and deep (59% and 60%) hematologic response rates between patients treated with daratumumab single agent and those who received daratumumab combinations.

Overall, the published trials and retrospective series indicated that daratumumab is highly effective also in relapsed/refractory patients with AL amyloidosis, with apparently no remarkable differences between single-agent and combination therapies.

### 4.3. Tolerability of Daratumumab in AL Amyloidosis

Our review of safety data of daratumumab in AL amyloidosis is based on prospective studies, due to possible underreporting of adverse events in retrospective series. Chemotherapy safety is an especially relevant issue in systemic AL amyloidosis because the hematologic malignancy needs to be effectively and rapidly targeted in patients with often severe amyloid-related multiorgan dysfunction and damage.

In the two phase II clinical trials in relapsed refractory patients, single-agent daratumumab was administered intravenously at the dosage of 16 mg/kg. In the study by Sanchorawala et al., grade 3 or 4 adverse events were observed in 91% of patients who received a median of 31 infusions [40]. The most common toxicities of any grade were respiratory illness (59%), iron deficiency (40%), infusion-related reaction (23%), atrial fibrillation (grade 3 or 4, 18%), and lymphopenia (14%) [40]. Only one patient stopped therapy due to a severe adverse event. A rigorous administration of pre- and post-infusion medication resulted in more manageable infusion-related reactions [40]. Roussel et al. reported grade 3 or 4 adverse events in 33% of patients who were treated for a median of 6 months (corresponding to 16 infusions) [41]. Overall, the most common adverse reactions of any grade were infections (55%), gastrointestinal disorders (42%), respiratory disorders (30%), fatigue (25%), and infusion-related reactions (13%) [41]. Remarkably, patients with cardiac involvement were able to tolerate intravenous infusions of daratumumab in this study [41]. The subcutaneous administration of daratumumab is expected to be associated with less frequent and less severe toxicity. In the ANDROMEDA phase III trial [36], grade 3 or 4 adverse events were reported in 43% of patients, and the most common adverse reactions of any grade were diarrhea (68%), fatigue (54%), peripheral edema (50%), upper respiratory tract infections (39%), and pneumonia (11%). In general, daratumumab-related toxicity is manageable in patients with AL amyloidosis, and administration of the monoclonal antibody is also feasible even intravenously in patients with cardiac involvement. Respiratory infections emerge as a common toxicity of this agent in AL amyloidosis, which should be particularly considered in fragile subjects and in those receiving prolonged treatment.

## 5. Conclusions and Perspectives

Targeting CD38 on plasma cells emerges as a new additional option in the treatment of AL amyloidosis. Daratumumab grants unprecedented high hematologic response rates both in combination with CyBorD in the upfront setting and as a rescue agent alone or in combination with bortezomib and lenalidomide. The recently completed randomized phase III ANDROMEDA trial has set daratumumab-bortezomib combinations as the new standard of care in AL amyloidosis. Moreover, while accessibility of daratumumab increases, many more daratumumab-naïve patients will receive this antibody as a rescue treatment. In the meantime, other CD38-targeting strategies are being tested in AL amyloidosis. For instance, the early results of a phase II trial (NCT03499808) of isatuximab (an IgG1κ chimeric monoclonal anti-CD38 antibody) in AL amyloidosis were presented at the 2020 meeting of the American Society of Hematology [61]. In 35 relapsed/refractory patients, isatuximab achieved an overall hematologic response rate of 77%, with 57% VGPR or better. Median time to a partial response (PR) or better was 1.1 months. One-year estimated PFS was 84%. lsatuximab treatment was well tolerated, and the most common grade ≥ 3 treatment-related adverse events (AEs) were lymphopenia in three patients (9%), lung infection in two patients (6%), and an infusion-related reaction in one patient (3%).

While the advent of daratumumab and possibly other CD38-targeting approaches will rapidly change the management of AL amyloidosis, the availability of this powerful agent is opening new challenges.

(1) In the relapsed/refractory setting, the efficacy of single-agent daratumumab does not appear to be lower than that of daratumumab combinations. However, it is not clear whether this also applies to treatment-naïve patients in the upfront setting when response rates have the greatest impact on survival. At present, we have no data to allow abandoning bortezomib in this setting in the general population, but single-agent daratumumab can be an appealing option in subjects with contraindications to bortezomib, such as peripheral neuropathy.
(2) Is maintenance with daratumumab needed in subjects who respond to daratumumab-CyBorD? In general, there are no data to support maintenance treatment in AL amyloidosis. This can be reasonable in patients who fulfil the criteria for multiple myeloma. However, unfortunately, the ANDROMEDA study was not designed to answer this question because all the patients enrolled in the daratumumab-CyBorD arm received maintenance with single-agent daratumumab.
(3) How should we treat patients who relapse after upfront daratumumab? If relapse occurs at a reasonable time after daratumumab discontinuation, rechallenge with the monoclonal antibody might be an option. Belantamab mafodotin is a first-in-class, BCMA-targeted antibody-drug conjugate that showed efficacy in myeloma patients relapsed/refractory to anti-CD38 antibodies [62]. A study of belantamab mafodotin in patients with relapsed or refractory AL amyloidosis (EMN27) is planned to start in 2021 (NCT04617925).
(4) Another rescue therapy in the future could be represented by the applicability of chimeric antigen receptor T cell (CAR-T) immunotherapies that have shown interesting results in relapsed/refractory MM. The feasibility of this novel treatment strategy in AL amyloidosis is already under evaluation and needs further investigations [63,64].

While the first phase III trials have just been completed in AL amyloidosis, we are understanding that our increased knowledge and our improved ability to treat patients are disclosing new needs and asking new complex questions that will require even larger trials. New studies are needed to address these relevant questions, and collaboration between academia and pharmaceutical industries will be necessary to design future trials.

## Figures and Tables

**Table 1:** Comparison of retrospective studies of daratumumab therapy in AL amyloidosis (only studies including ≥ 10 patients are reported).

Reference	Treatment Regimen, Number of Evaluable Patients (% of the Total)	N. of Previous Lines of Therapy, Median (Range)	Hematologic Response, % (CR, VGPR)	Organ Response
Khouri et al. [45]	Mono, 15 (100)	3 (1–10)	86 (33/53)	NA
Abeykoon et al. [46]	Mono, 14 (46) Combo, 16 (54)	3 (1–8)	78 (14/64) 88 (19/63)	Cardiac 44% Renal 27%
Schwotzer et al. [51]	Mono, 10 (100)	3 (1–5)	90 (40/30)	Cardia 50%
Godara et al. [53]	Mono, 10 (53) Combo, 9 (47) *	1 (1–5)	[80, CR+VGPR] [100, CR+VGPR]	Cardiac 78% Renal 66%
Chung et al. [54]	Mono, 52 (100)	2 (1–3)	77 (40/30)	Cardiac 55% Renal 52%
Lecumberri et al. [55]	Mono, 36 (100)	2 (1–8)	72 (28/36)	Cardiac 37% Renal 59%
Kimmich et al. [56] **	Mono, 92 (55) Combo, 53 (45)	2 (1–7)	64 (8/47) 66 (11/54)	Cardiac 22%, Renal 24% Cardiac 28%, Renal 27%
Milani et al. [57]	Mono, 47 (65) Combo, 25 (35)	2 (1–9)	80 (29/29) 88 (18/28)	Cardiac 29% Renal 60%
Cohen et al. [58]	Mono, 50 (100)	3 (1–4)	84 (38/28)	Cardiac 74% Renal 73%
Shragai et al. [60]	Mono, 19 (38) Combo, 29 (62)	1 (1–6)	81 (≥VGPR 64)	Cardiac 74% Renal 73%

* Nine patients were treated with MoAb NEOD001. ** Kimmich et al. reported proportion of organ responders over time, rather than response at any time after daratumumab. Mono, daratumumab monotherapy; Combo, daratumumab combinations with lenalidomide or bortezomib; CR, complete response; NA, not available; VGPR, very good partial response; “Cardiac” response, according to Palladini et al. [7]; “Renal” response, according to Palladini et al. [8].

## References

[1] Merlini G., Stone M. (2006). Dangerous small B-cell clones. Blood.

[2] Merlini G., Dispenzieri A., Sanchorawala V., Schonland S.O., Palladini G., Hawkins P.N., Gertz M.A. (2018). Systemic immunoglobulin light chain amyloidosis. Nat. Rev. Dis. Primers.

[3] Palladini G., Milani P., Merlini G. (2020). Management of AL amyloidosis in 2020. Blood.

[4] Godara A., Palladini G. (2020). Monoclonal Antibody Therapies in Systemic Light-Chain Amyloidosis. Hematol. Oncol. Clin. N. Am..

[5] Palladini G., Lavatelli F., Russo P., Perlini S., Perfetti V., Bosoni T., Obici L., Bradwell A., D’Eril G., Fogari R. (2006). Circulating amyloidogenic free light chains and serum N-terminal natriuretic peptide type B decrease simultaneously in association with improvement of survival in AL. Blood.

[6] Manwani R., Foard D., Mahmood S., Sachchithanantham S., Lane T., Quarta C., Youngstein T., Rezk T., Lachmann H.J., Gillmore J.D. (2018). Rapid hematologic responses improve outcomes in patients with very advanced (stage IIIb) cardiac immunoglobulin light chain amyloidosis. Haematologica.

[7] Palladini G., Dispenzieri A., Gertz M.A., Kumar S., Wechalekar A., Hawkins P.N., Schonland S., Hegenbart U., Comenzo R., Kastritis E. (2012). New criteria for response to treatment in immunoglobulin light chain amyloidosis based on free light chain measurement and cardiac biomarkers: Impact on survival outcomes. J. Clin. Oncol..

[8] Palladini G., Hegenbart U., Milani P., Kimmich C., Foli A., Ho A.D., Vidus Rosin M., Albertini R., Moratti R., Merlini G. (2014). A staging system for renal outcome and early markers of renal response to chemotherapy in AL amyloidosis. Blood.

[9] Palladini G., Schonland S.O., Sanchorawala V., Kumar S., Wechalekar A., Hegenbart U., Milani P., Ando Y., Westermark P., Dispenzieri A. (2021). Clarification on the definition of complete haematologic response in light-chain (AL) amyloidosis. Amyloid.

[10] Sidana S., Larson D.P., Greipp P.T., He R., McPhail E.D., Dispenzieri A., Murray D.L., Dasari S., Ansell S.M., Muchtar E. (2020). IgM AL amyloidosis: Delineating disease biology and outcomes with clinical, genomic and bone marrow morphological features. Leukemia.

[11] Sachchithanantham S., Roussel M., Palladini G., Klersy C., Mahmood S., Venner C.P., Gibbs S., Gillmore J., Lachmann H., Hawkins P.N. (2016). European Collaborative Study Defining Clinical Profile Outcomes and Novel Prognostic Criteria in Monoclonal Immunoglobulin M-Related Light Chain Amyloidosis. J. Clin. Oncol..

[12] Basset M., Defrancesco I., Milani P., Nuvolone M., Rattotti S., Foli A., Mangiacavalli S., Varettoni M., Benvenuti P., Cartia C.S. (2020). Nonlymphoplasmacytic lymphomas associated with light-chain amyloidosis. Blood.

[13] Bochtler T., Merz M., Hielscher T., Granzow M., Hoffmann K., Kramer A., Raab M.S., Hillengass J., Seckinger A., Kimmich C. (2018). Cytogenetic intraclonal heterogeneity of plasma cell dyscrasia in AL amyloidosis as compared with multiple myeloma. Blood Adv..

[14] Paiva B., Martinez-Lopez J., Corchete L.A., Sanchez-Vega B., Rapado I., Puig N., Barrio S., Sanchez M.L., Alignani D., Lasa M. (2016). Phenotypic, transcriptomic, and genomic features of clonal plasma cells in light-chain amyloidosis. Blood.

[15] Cuenca I., Alameda D., Sanchez-Vega B., Gomez-Sanchez D., Alignani D., Lasa M., Onecha E., Lecumberri R., Prosper F., Ocio E.M. (2021). Immunogenetic characterization of clonal plasma cells in systemic light-chain amyloidosis. Leukemia.

[16] Boyle E.M., Ashby C., Wardell C.P., Rowczenio D., Sachchithanantham S., Wang Y., Johnson S.K., Bauer M.A., Weinhold N., Kaiser M.F. (2018). The genomic landscape of plasma cells in systemic light chain amyloidosis. Blood.

[17] Huang X.F., Jian S., Lu J.L., Shen K.N., Feng J., Zhang C.L., Tian Z., Wang J.L., Lei W.J., Cao X.X. (2020). Genomic profiling in amyloid light-chain amyloidosis reveals mutation profiles associated with overall survival. Amyloid.

[18] Da Silva Filho M.I., Forsti A., Weinhold N., Meziane I., Campo C., Huhn S., Nickel J., Hoffmann P., Nothen M.M., Jockel K.H. (2017). Genome-wide association study of immunoglobulin light chain amyloidosis in three patient cohorts: Comparison with myeloma. Leukemia.

[19] Chattopadhyay S., Thomsen H., Weinhold N., Meziane I., Huhn S., da Silva Filho M.I., Vodicka P., Vodickova L., Hoffmann P., Nothen M.M. (2020). Eight novel loci implicate shared genetic etiology in multiple myeloma, AL amyloidosis, and monoclonal gammopathy of unknown significance. Leukemia.

[20] Oliva L., Orfanelli U., Resnati M., Raimondi A., Orsi A., Milan E., Palladini G., Milani P., Cerruti F., Cascio P. (2017). The amyloidogenic light chain is a stressor that sensitizes plasma cells to proteasome inhibitor toxicity. Blood.

[21] Bochtler T., Hegenbart U., Kunz C., Granzow M., Benner A., Seckinger A., Kimmich C., Goldschmidt H., Ho A.D., Hose D. (2015). Translocation t(11;14) is associated with adverse outcome in patients with newly diagnosed AL amyloidosis when treated with bortezomib-based regimens. J. Clin. Oncol..

[22] Muchtar E., Dispenzieri A., Kumar S.K., Ketterling R.P., Dingli D., Lacy M.Q., Buadi F.K., Hayman S.R., Kapoor P., Leung N. (2017). Interphase fluorescence in situ hybridization in untreated AL amyloidosis has an independent prognostic impact by abnormality type and treatment category. Leukemia.

[23] Bochtler T., Hegenbart U., Kunz C., Benner A., Kimmich C., Seckinger A., Hose D., Goldschmidt H., Granzow M., Dreger P. (2016). Prognostic impact of cytogenetic aberrations in AL amyloidosis patients after high-dose melphalan: A long-term follow-up study. Blood.

[24] Bryce A.H., Ketterling R.P., Gertz M.A., Lacy M., Knudson R.A., Zeldenrust S., Kumar S., Hayman S., Buadi F., Kyle R.A. (2009). Translocation t(11;14) and survival of patients with light chain (AL) amyloidosis. Haematologica.

[25] Warsame R., Kumar S.K., Gertz M.A., Lacy M.Q., Buadi F.K., Hayman S.R., Leung N., Dingli D., Lust J.A., Ketterling R.P. (2015). Abnormal FISH in patients with immunoglobulin light chain amyloidosis is a risk factor for cardiac involvement and for death. Blood Cancer J..

[26] Malavasi F., Deaglio S., Funaro A., Ferrero E., Horenstein A.L., Ortolan E., Vaisitti T., Aydin S. (2008). Evolution and function of the ADP ribosyl cyclase/CD38 gene family in physiology and pathology. Physiol. Rev..

[27] Horenstein A.L., Bracci C., Morandi F., Malavasi F. (2019). CD38 in Adenosinergic Pathways and Metabolic Re-programming in Human Multiple Myeloma Cells: In-tandem Insights from Basic Science to Therapy. Front. Immunol..

[28] Martin T.G., Corzo K., Chiron M., Velde H.V., Abbadessa G., Campana F., Solanki M., Meng R., Lee H., Wiederschain D. (2019). Therapeutic Opportunities with Pharmacological Inhibition of CD38 with Isatuximab. Cells.

[29] Seckinger A., Hillengass J., Emde M., Beck S., Kimmich C., Dittrich T., Hundemer M., Jauch A., Hegenbart U., Raab M.S. (2018). CD38 as Immunotherapeutic Target in Light Chain Amyloidosis and Multiple Myeloma-Association With Molecular Entities, Risk, Survival, and Mechanisms of Upfront Resistance. Front. Immunol..

[30] Van de Donk N., Richardson P.G., Malavasi F. (2018). CD38 antibodies in multiple myeloma: Back to the future. Blood.

[31] Krejcik J., Casneuf T., Nijhof I.S., Verbist B., Bald J., Plesner T., Syed K., Liu K., van de Donk N.W., Weiss B.M. (2016). Daratumumab depletes CD38+ immune regulatory cells, promotes T-cell expansion, and skews T-cell repertoire in multiple myeloma. Blood.

[32] Viola D., Dona A., Caserta E., Troadec E., Besi F., McDonald T., Ghoda L., Gunes E.G., Sanchez J.F., Khalife J. (2021). Daratumumab induces mechanisms of immune activation through CD38+ NK cell targeting. Leukemia.

[33] Atanackovic D., Yousef S., Shorter C., Tantravahi S.K., Steinbach M., Iglesias F., Sborov D., Radhakrishnan S.V., Chiron M., Miles R. (2020). In vivo vaccination effect in multiple myeloma patients treated with the monoclonal antibody isatuximab. Leukemia.

[34] Administration U.S.F.D. FDA Grants Accelerated Approval to Darzalex Faspro for Newly Diagnosed Light Chain Amyloidosis. https://www.fda.gov/drugs/drug-approvals-and-databases/fda-grants-accelerated-approval-darzalex-faspro-newly-diagnosed-light-chain-amyloidosis.

[35] Palladini G., Kastritis E., Maurer M.S., Zonder J., Minnema M.C., Wechalekar A.D., Jaccard A., Lee H.C., Bumma N., Kaufman J.L. (2020). Daratumumab plus CyBorD for patients with newly diagnosed AL amyloidosis: Safety run-in results of ANDROMEDA. Blood.

[36] Kastritis E., Palladini G., Minnema M., Wechalekar A., Jaccard A., Lee H., Sanchorawala V., Gibbs S., Mollee P., Venner C. Subcutaneous Daratumumab + Cyclophosphamide, Bortezomib, and Dexamethasone (CYBORD) in Patients with Newly Diagnosed Light Chain (AL) Amyloidosis: Primary Results from the Phase 3 ANDROMEDA Study. EHA Meeting 2020, 13 June 2020. https://ehaweb.org/meetings/.

[37] Gertz M., Comenzo R., Falk R., Fermand J., Hazenberg B., Hawkins P., Merlini G., Moreau P., Ronco P., Sanchorawala V. (2005). Definition of organ involvement and treatment response in immunoglobulin light chain amyloidosis (AL): A consensus opinion from the 10th International Symposium on Amyloid and Amyloidosis, Tours, France, 18–22 April 2004. Am. J. Hematol..

[38] Comenzo R., Kastritis E., Palladini G., Minnema M., Wechalekar A., Jaccard A., Sanchorawala V., Lee H., Gibbs S., Mollee P. (2020). Reduction in Absolute Involved Free Light Chain and Difference between Involved and Uninvolved Free Light Chain Is Associated with Prolonged Major Organ Deterioration Progression-Free Survival in Patients with Newly Diagnosed AL Amyloidosis Receiving Bortezomib, Cyclophosphamide, and Dexamethasone with or without Daratumumab: Results from Andromeda. Blood.

[39] Wechalekar A., Palladini G., Merlini G., Comenzo R., Jaccard A., Tran N., Pei H., Vasey S., Tromp B., Weiss B. (2020). Rapid and Deep Hematologic Responses Are Associated with Improved Major Organ deterioration Progression-Free Survival in Newly Diagnosed AL Amyloidosis: Results from Andromeda. Blood.

[40] Sanchorawala V., Sarosiek S., Schulman A., Mistark M., Migre M.E., Cruz R., Sloan J.M., Brauneis D., Shelton A.C. (2020). Safety, tolerability, and response rates of daratumumab in relapsed AL amyloidosis: Results of a phase 2 study. Blood.

[41] Roussel M., Merlini G., Chevret S., Arnulf B., Stoppa A.M., Perrot A., Palladini G., Karlin L., Royer B., Huart A. (2020). A prospective phase 2 trial of daratumumab in patients with previously treated systemic light-chain amyloidosis. Blood.

[42] Sher T., Fenton B., Akhtar A., Gertz M.A. (2016). First report of safety and efficacy of daratumumab in 2 cases of advanced immunoglobulin light chain amyloidosis. Blood.

[43] Kaufman G.P., Schrier S.L., Lafayette R.A., Arai S., Witteles R.M., Liedtke M. (2017). Daratumumab yields rapid and deep hematologic responses in patients with heavily pretreated AL amyloidosis. Blood.

[44] Gran C., Gahrton G., Alici E., Nahi H. (2018). Case Report: Treatment of light-chain amyloidosis with daratumumab monotherapy in two patients. Eur. J. Haematol..

[45] Khouri J., Kin A., Thapa B., Reu F.J., Bumma N., Samaras C.J., Liu H.D., Karam M.A., Reed J., Mathur S. (2019). Daratumumab proves safe and highly effective in AL amyloidosis. Br. J. Haematol..

[46] Abeykoon J.P., Zanwar S., Dispenzieri A., Gertz M.A., Leung N., Kourelis T., Gonsalves W., Muchtar E., Dingli D., Lacy M.Q. (2019). Daratumumab-based therapy in patients with heavily-pretreated AL amyloidosis. Leukemia.

[47] Arnall J.R., Usmani S.Z., Adamu H., Mishkin J., Bhutani M. (2019). Daratumumab, pomalidomide, and dexamethasone as a bridging therapy to autologous stem cell transplantation in a case of systemic light-chain amyloidosis with advanced cardiac involvement. J. Oncol. Pharm. Pract..

[48] Canichella M., Serrao A., Annechini G., D’Elia G.M., De Luca M.L., Pulsoni A. (2019). Long-term response to daratumumab in a patient with advanced immunoglobulin light-chain (AL) amyloidosis with organ damage. Ann. Hematol..

[49] Lee L.X., Zhou P., Varga C., Fogaren T., Ho K., Ma X., Warner M., Toskic D., Kugelmass A., Comenzo R.L. (2019). Daratumumab activity in relapsed or primary refractory systemic AL amyloidosis and Fcγ receptor 3A V158F polymorphisms. Amyloid.

[50] Ghilardi G., Stussi G., Mazzucchelli L., Röcken C., Rossi D., Gerber B. (2019). Venetoclax plus daratumumab induce hematological CR and organ response in an AL amyloidosis patient with t(11;14). Amyloid.

[51] Schwotzer R., Manz M.G., Pederiva S., Waibel C., Caspar C., Lerch E., Flammer A.J., Brouwers S., Seeger H., Heimgartner R. (2019). Daratumumab for relapsed or refractory AL amyloidosis with high plasma cell burden. Hematol. Oncol..

[52] Van de Wyngaert Z., Carpentier B., Pascal L., Lionne-Huyghe P., Leduc I., Srour M., Vasseur M., Demarquette H., Terriou L., Herbaux C. (2020). Daratumumab is effective in the relapsed or refractory systemic light-chain amyloidosis but associated with high infection burden in a frail real-life population. Br. J. Haematol..

[53] Godara A., Siddiqui N.S., Lee L.X., Toskic D., Fogaren T., Varga C., Comenzo R.L. (2020). Dual Monoclonal Antibody Therapy in Patients With Systemic AL Amyloidosis and Cardiac Involvement. Clin. Lymphoma Myeloma Leuk..

[54] Chung A., Kaufman G.P., Sidana S., Eckhert E., Schrier S.L., Lafayette R.A., Arai S., Witteles R.M., Liedtke M. (2020). Organ responses with daratumumab therapy in previously treated AL amyloidosis. Blood Adv..

[55] Lecumberri R., Krsnik I., Askari E., Sirvent M., González-Pérez M.S., Escalante F., Pradillo V., Tamariz L.E., Cánovas V., Alegre A. (2020). Treatment with daratumumab in patients with relapsed/refractory AL amyloidosis: A multicentric retrospective study and review of the literature. Amyloid.

[56] Kimmich C.R., Terzer T., Benner A., Dittrich T., Veelken K., Carpinteiro A., Hansen T., Goldschmidt H., Seckinger A., Hose D. (2020). Daratumumab for systemic AL amyloidosis: Prognostic factors and adverse outcome with nephrotic-range albuminuria. Blood.

[57] Milani P., Fazio F., Basset M., Berno T., Larocca A., Foli A., Riva M., Benigna F., Oliva S., Nuvolone M. (2020). High rate of profound clonal and renal responses with daratumumab treatment in heavily pre-treated patients with light chain (AL) amyloidosis and high bone marrow plasma cell infiltrate. Am. J. Hematol..

[58] Cohen O.C., Brodermann M.H., Blakeney I.J., Mahmood S., Sachchithanantham S., Ravichandran S., Law S., Lachmann H.J., Whelan C.J., Popat R. (2020). Rapid response to single agent daratumumab is associated with improved progression-free survival in relapsed/refractory AL amyloidosis. Amyloid.

[59] Roccatello D., Fenoglio R., Naretto C., Baldovino S., Sciascia S., Ferro M., Rossi D. (2020). Daratumumab Monotherapy in Severe Patients with AL Amyloidosis and Biopsy-Proven Renal Involvement: A Real Life Experience. J. Clin. Med..

[60] Shragai T., Gatt M., Lavie N., Vaxman I., Tadmor T., Rouvio O., Zektser M., Horowitz N., Magen H., Ballan M. (2020). Daratumumab for relapsed AL amyloidosis—When cumulative real-world data precedes clinical trials: A multisite study and systematic literature review. Eur. J. Haematol..

[61] Parker T., Rosenthal A., Sanchorawala V., Landau H., Campagnaro E., Kapoor P., Neparidze N., Hagen P., Sarosiek S., Scott E. (2020). A Phase II Study of Isatuximab (SAR650984) NSC-795145) for Patients with Previously Treated AL Amyloidosis (SWOGS1702; NCT#03499808). Blood.

[62] Lonial S., Lee H.C., Badros A., Trudel S., Nooka A.K., Chari A., Abdallah A.O., Callander N., Lendvai N., Sborov D. (2020). Belantamab mafodotin for relapsed or refractory multiple myeloma (DREAMM-2): A two-arm, randomised, open-label, phase 2 study. Lancet Oncol..

[63] Rosenzweig M., Urak R., Walter M., Lim L., Sanchez J.F., Krishnan A., Forman S., Wang X. (2017). Preclinical data support leveraging CS1 chimeric antigen receptor T-cell therapy for systemic light chain amyloidosis. Cytotherapy.

[64] Zmievskaya E., Valiullina A., Ganeeva I., Petukhov A., Rizvanov A., Bulatov E. (2021). Application of CAR-T Cell Therapy beyond Oncology: Autoimmune Diseases and Viral Infections. Biomedicines.

